# VO2MAX, 6-minute walk, and muscle strength each correlate with frailty in US veterans

**DOI:** 10.3389/fphys.2024.1393221

**Published:** 2024-09-13

**Authors:** Kenneth Ladd Seldeen, Ayesha Saqebur Rahman, Yonas Redae, Nikhil Satchidanand, M. Jeffery Mador, Changxing Ma, Mihir Soparkar, Alexis Rose Lima, Ifeoma N. Ezeilo, Bruce Robert Troen

**Affiliations:** ^1^ Division of Geriatrics, Department of Internal Medicine and Landon Center on Aging, University at Kansas Medical Center, Kansas City, KS, United States; ^2^ Research Service, VA Kansas City Healthcare System, Kansas City, MO, United States; ^3^ Research Service, VA Western New York Healthcare System, Buffalo, NY, United States; ^4^ Division of Geriatrics and Palliative Medicine, Department of Medicine, Jacobs School of Medicine and Biomedical Sciences, University at Buffalo, Buffalo, NY, United States; ^5^ Division of Pulmonary, Critical Care, and Sleep Medicine, Department of Medicine, Jacobs School of Medicine and Biomedical Sciences, University at Buffalo, Buffalo, NY, United States; ^6^ Department of Biostatistics, School of Public Health and Health Professions, Buffalo, NY, United States

**Keywords:** frailty, VO2max, 6-minute walk, inflammation, short physical performance battery (SPPB)

## Abstract

**Introduction:**

Frailty often manifests as an increased vulnerability to adverse outcomes, and detecting frailty is useful for informed healthcare decisions. Veterans are at higher risk for developing frailty and at younger ages. The goal of this study was to investigate approaches in Veterans that can better inform the physiologic underpinnings of frailty, including maximal oxygen uptake (VO2max), 6-min walk, muscle strength, and inflammatory biomarkers.

**Methods:**

Participants (N = 42) were recruited from the Buffalo VA Medical Center. Inclusion criteria: ages 60–85, male or female, any race, and not having significant comorbidities or cognitive impairment. Outcome measures included: the Fried frailty phenotype, the short physical performance battery (SPPB), quality of life (QOL) using the Q-LES-Q-SF, and the following physiologic assessments: VO2max assessment on an upright stationary bicycle, 6-min walk, and arm and leg strength. Additionally, inflammatory biomarkers (C-reactive protein, IL-6, IL-10, interferon-γ, and TNF-α) were measured using ELLA single and multiplex ELISA.

**Results:**

Participants: 70.3 ± 7.4 years of age: 34 males and 8 females, BMI = 30.7 ± 5.4 kg/m^2^, 26 white and 16 African American. A total of 18 (42.8%) were non-frail, 20 (47.6%) were pre-frail, and 4 (9.5%) were frail. VO2max negatively correlated with Fried frailty scores (r = −0.40, p = 0.03, N = 30), and positively correlated with SPPB scores (r = 0.50, p = 0.005), and QOL (r = 0.40, p = 0.03). The 6-min walk test also significantly correlated with VO2max (r = 0.57, p = 0.001, N = 42) and SPPB (r = 0.55, p = 0.0006), but did not quite reach a significant association with frailty (r = −0.28, p = 0.07). Arm strength negatively correlated with frailty (r = −0.47, p = 0.02, N = 26), but not other parameters. Inflammatory profiles did not differ between non-frail and pre-frail/frail participants.

**Conclusion:**

Objectively measured cardiorespiratory fitness was associated with important functional outcomes including physical performance, QOL, and frailty in this group of older Veterans. Furthermore, the 6-min walk test correlated with VO2max and SPPB, but more validation is necessary to confirm sensitivity for frailty. Arm strength may also be an important indicator of frailty, however the relationship to other indicators of physical performance is unclear.

## 1 Introduction

In the United States more than 30% of Veterans 65 years or older are frail, which is three-times higher than in non-Veteran Americans of the same age (Orkaby et al., 2018). Further, prevalence of frailty increases with age, impacting 20% of all adults 70–79 years old and 50% of adults 85 and over (Xue, 2011; Clegg et al., 2013). Frail individuals have greater susceptibility to stressors that leads to adverse outcomes including falls, disability, hospitalization, and mortality (Clegg et al., 2013; Bandeen-Roche et al., 2015; Bandeen-Roche et al., 2006; Chang et al., 2019; Fried et al., 2001). Given these important associations, frailty determination is emerging as a useful tool to predict future adverse events, particularly before surgery (Gillis et al., 2022; Gritsenko et al., 2020). Furthermore, identifying new approaches to measure and correlate with frailty can be clinically useful by informing the physiologic underpinnings of frailty and the potential for providing predictive biomarkers.

Toward this goal, others have investigated single assessments that correlate with physical frailty. For example, grip strength measurement has been found to correlate with frailty, suggesting a quick alternative (Dudzinska-Griszek et al., 2017; Reeve et al., 2018; Spiegowski et al., 2022; Syddall et al., 2003). However, there may be sex specific confounders that affect the predictive value of grip strength (Spiegowski et al., 2022), but this surrogate measure did not predict mortality (Dudzinska-Griszek et al., 2017), for which frailty is a predictor (Fried et al., 2001). Likewise, gait speed assessment (typically over 4 m) has also been correlated with frailty and may be a promising single assessment alternative (Clegg et al., 2015; Pamoukdjian et al., 2015; Schoon et al., 2014) – although both grip strength and gait speed are parameters of the Fried frailty assessment tool (Fried et al., 2001). Finally, there is growing interest in identifying blood biomarkers, including inflammatory cytokines, for detection of frailty and other age-related declines (Picca et al., 2022). Although these relationships have not been examined in older Veterans, relationships have been identified with C-reactive protein and interleukin-6 and frailty (Soysal et al., 2016).

Here we investigate the relationship between frailty and three physical performance assessments - maximal exercise capacity (VO2max), 6-min walk test, and muscle strength – in a US Veteran cohort. There is a paucity of frailty investigation into Veteran populations, which are at greater risk of frailty and subsequent disability, morbidity, and mortality (Ganta et al., 2021; Orkaby et al., 2019). VO2max assessment is a gold standard test to evaluate cardiovascular fitness (Laukkanen et al., 2001), however to our knowledge its relationship with frailty in Veterans has not been examined. Likewise, 6-min walk performance has been correlated to frailty status (Boxer et al., 2010; Boxer et al., 2008), but not in Veteran populations. Furthermore, as discussed previously, grip strength as determined by hand dynamometers has also been correlated with frailty (Dudzinska-Griszek et al., 2017; Reeve et al., 2018; Spiegowski et al., 2022; Buckinx et al., 2019; Karagiannopoulos et al., 2022; Vaidya et al., 2018), however, here we examine for the first time if arm and leg strength correlate with frailty. To investigate this knowledge gap, we therefore measured frailty and its relationship to functional capacity in older US Veterans.

## 2 Materials and methods

### 2.1 Study population

Participants for this study were initially recruited for an exercise trial (NCT03750006), and the study herein reports baseline data. The enrollees included male and female United States Veterans between the ages of 60–85 years of age and of any race. Participants were excluded if they exhibited severe comorbidities [cardiac disease (≥ class III), chronic obstructive pulmonary disease (severe as determined by an FEV1 below 50%), chronic kidney disease (≥ stage 3)], VA – St. Louis University Mental Status (VA-SLUMS) cognitive score less than 20, or could not operate a stationary exercise bike. Participants were recruited from the greater Buffalo, NY area. The study was approved by the VA Western New York Internal Review Board with protocol number #1580041.

### 2.2 Surveys

Surveys were administered in an interview style including the VA St. Louis University Mental Survey (VA-SLUMS, (Arndt, 2006)) and the Quality of Life, Satisfaction, and Enjoyment Short Form (Q-LES-Q-SF, (Endicott et al., 1993)).

### 2.3 Functional assessment testing

#### 2.3.1 Maximal exercise testing (VO2max)

An incremental symptom-limited exercise test was performed on an electronically braked cycle ergometer (Corival CPET ergometer, MGC diagnostics, St. Paul Minnesota, United States) to determine each participant’s VO2max. After 1 min of pedaling at 0 W, the workload was increased by 15–20 W every minute until the participant could no longer continue. The chosen increment difference was determined on the participants response to the question, “are you physical active and/or can you walk a mile without difficulty.” If the answer was “yes,” then the increment employed was 20 W, if “no,” then the increment was 15 W. The last workload for which a participant was able to complete 30 s of cycling was designated as maximum work capacity during which VO2max was calculated. Oxygen consumption was measured using a metabolic exercise cart (Ultima Cardi O2, St. Paul Minnesota, United States).

#### 2.3.2 6-Minute walk

Walk endurance was determined by asking participants to walk with a “constant and brisk pace” back and forth on a 50-foot (15.24 m) straight track for 6 total minutes. Standard instructions were given to all participants.

#### 2.3.3 Grip strength

Maximal grip strength was assessed using a hydraulic hand dynamometer (JLW Instruments, Chicago, IL) as the best of three trials using the dominant hand. Each trial consisted of squeezing the instrument for a total of 5 s.

#### 2.3.4 Muscle strength

Leg and arm muscle strength were measured using a microFET2 dynamometer (Hoggan Scientific, LLC, Salt Lake City, UT) as the best of 3 trials for each limb, and has been previously validated and used in older adults (Buckinx et al., 2019; Karagiannopoulos et al., 2022; Vaidya et al., 2018). For leg strength (quadriceps muscle) trials, the participant was seated with the leg positioned with a knee bent at 90°. The dynamometer was then held in a supported fashion just above the ankle, and the participant attempted to extend the lower leg forward for 5 s. For the arm (biceps muscle) trial, the arm was extended, and the dynamometer placed on the wrist, holding the arm down as the participant was asked to perform an arm curl for 5 s.

#### 2.3.5 Gait speed

Gait speed was assessed by having participants walk with a “usual walking speed” pace for a total of 10 feet (3.05 m, for use in short physical performance battery) and then 15 feet (4.57 m, for use in frailty assessment), with a 5 foot (1.52 m) acceleration zone before the start point.

#### 2.3.6 Short physical performance battery (SPPB)

The SPPB consisted of a combined score of three assessments (max score 4 points for each for 12 total points) that included balance, chair rise, and gait speed (Guralnik et al., 1994). For the balance test, participants were asked to stand feet together, feet off-set with the heel of one foot being approximately lined up with the midpoint of the other foot (semi-tandem), and foot in front of the other foot for a total of 10 s per stance (tandem). Scoring for balance was 0 for unable or 0–9 s for feet together, 1 for feet together 10 s but <10 s on semi-tandem, 2 if semi-tandem 10 s, but tandem 0–2 s, 3 if semi-tandem 10 s, but tandem 3–9 s, and 4 if tandem is 10 s. For chair rise, the participant was asked to rise “as briskly as you feel safe enough to do so” pace and timed for 5 total chair rises. Participants were timed from the point of initiating the first rise to completing the last rise. Chair rise was scored as 0 for unable, 1 for >16.7 s, 2 for 13.7–16.6 s, 3 for 11.2–13.6 s, and 4 for ≤11.1 s. For gait speed, the time to complete the 10-foot walk was used for calculation of total SPPB score as 0 if could not do, 1 if <0.43 m/s, 2 if between 0.44–0.60 m/s, 3 if between 0.61–0.77 m/s, and 4 if gait speed was >0.78 m/s.

### 2.4 Frailty assessment

Frailty was assessed as the combined score of 5 different parameters as per (Drey et al., 2011), including: self-reported unexpected weight loss with >10 pounds or ≥5% of loss in a 1 year period; low activity assessed by self-report of not engaging in either moderate or vigorous activity; poor endurance with self-report of feeling “not full of energy” or spending >1 h in bed during the day more than once per week; weak grip strength defined as ≤ 29 kg for BMI <24 kg/m^2^, ≤30 kg for BMI between 24 and 28 kg/m^2^, and ≤32 kg for BMI >28 kg/m^2^ for men, and as ≤17 kg for BMI <23 kg/m^2^, ≤18 kg for BMI between 23 and 29 kg/m^2^, and ≤21 kg for BMI >29 kg/m^2^ for women; and slow gait speed defined as ≥7 s to walk 4 m for a height ≤ 1.73 m or ≥6 s for a height >1.73 m for men, and as ≥ 7 s to walk 4 m for a height ≤ 1.59 m or ≥6 s for a height >1.59 m for women (Karagiannopoulos et al., 2022).

### 2.5 Serum inflammatory cytokines

Blood was collected following an overnight fast and between the hours of 8 a.m. and 11 a.m. for all participants. Samples were centrifuged at 2,000 RPM for 10 min using a Beckman Allegra 6R refrigerated benchtop centrifuge allowing collection and aliquoting of serum. Cytokines were measured using a Bio-Techne ELLA multiplex ELISA system (Minneapolis, MN) and either custom single plex ELISA plates for human C-reactive protein, or multiplexed plates for interleukin-6, interleukin-10, interferon gamma, and tumor necrosis factor alpha from Bio-Techne. Analysis of plates was performed using Bio-Techne ELLA system software.

### 2.6 Statistics

A combination of descriptive statistics, chi-square test (Fisher’s exact test), and Pearson’s correlations was used to analyze relationships between frailty, functional assessments, and survey data. Continuous variables were expressed as mean ± SD and categorical variables as frequencies. Additionally, serum inflammatory cytokines were assessed using an unpaired student’s T-test comparing participants categorized as non-frail (frailty score of 0) with those that were either prefrail (score 1 or 2) or frail (score 3 or greater) per (Fried et al., 2001). Additionally, we performed a Shapiro-Wilks test and found our outcome measures do not contradict the normality assumption and therefore we did not use a non-parametric approach. We also did not investigate the potential for confounding variables due to small sample sizes. We estimated 80% power using a significance level of 0.05 and given a medium correlation coefficient of 0.40 and a sample size of 34 participants. A p-value <0.05 was considered significant. All analyses were performed using SAS 9.4 (SAS Institute Inc., Cary, NC).

## 3 Results

### 3.1 Prefrailty was prevalent in this veteran cohort and correlated with lower quality of life

**FIGURE 1:**
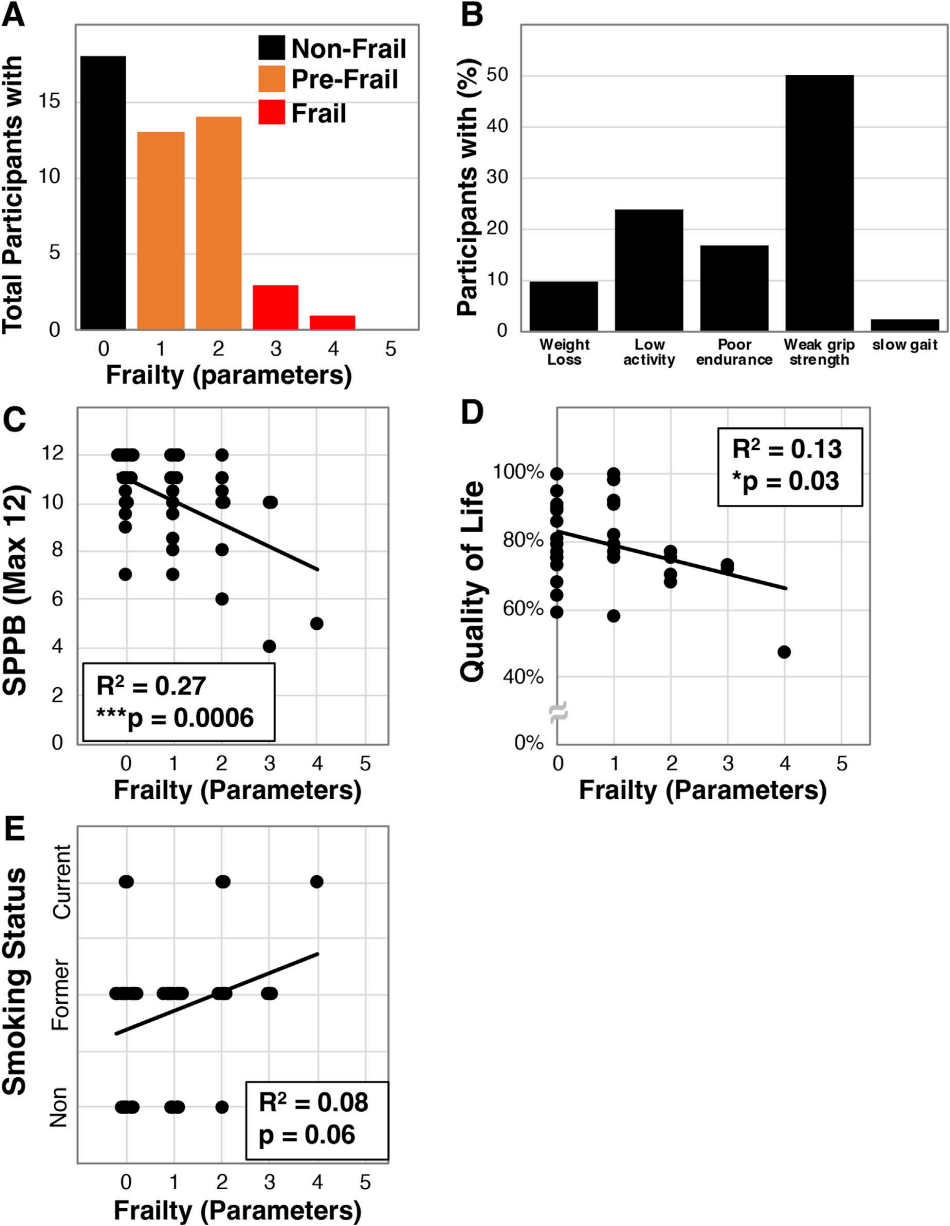
Frailty characterization of older Veterans. Frailty was assessed using the Fried *et al.* physical frailty phenotype in a total of 42 older Veterans allowing for determination of the number of participants with specific parameters **(A)** and the percentage of Veterans having each parameter **(B)**. Additionally, frailty in this cohort was correlated with disability measured by the SPPB **(C)**, the quality of life survey - Q-LES-Q-SF **(D)**, and smoking status **(E)**. Statistical significance indicated by “*” indicates p < 0.05 and “***” indicates p < 0.001.

We investigated frailty in community dwelling older Veterans (average age 70.3 ± 7.4, N = 42, Table 1) using baseline data collected for participants we enrolled into an exercise intervention study. The population was generally male (81%), either Caucasian (62%) or African American (38%), had a high BMI (30.7 ± 5.4 kg/m^2^), and most were a former or present smoker (62% and 12% respectively). Within this population we identified that 18 of the 42 were non-frail (42.9%), 20 pre-frail (47.6%), and 4 frail (9.5%, Figure 1A). Additionally, 50.0% of the participants were below the parameter cut-off for grip strength while only 1 (2.4%) was below cut-off for gait speed (Figure 1B). We also identified that frailty in these Veterans correlated with disability as assessed by the short physical performance battery (SPPB, R^2^ = 0.27, ***p = 0.0006, Figure 1C). Furthermore, we examined whether frailty associates with quality of life using the Quality of Life, Enjoyment, and Satisfaction Questionnaire Short Form [Q-LES-Q-SF, (Endicott et al., 1993)] - a 14-item survey that examines self-rated perceptions of functional capacity, social interactions, housing, and financial wellbeing among other topics. We found that greater frailty negatively correlated with quality of life in this population (R^2^ = 0.13, *p = 0.03, Figure 1D). Finally, we investigated the relationship with smoking history and found a trend towards frailty with increased usage (R^2^ = 0.08, p = 0.06, Figure 1E).

**TABLE 1:** Characteristics of participants.

*Characteristic*	N *=* 42
Sex, *n* (%)	
Male	34 (81%)
Female	8 (19%)
Age, years, mean (SD), range	70.3 (±7.4), 60–85
Race, *n* (%)	
Caucasian	26 (62%)
African American	16 (38%)
BMI, kg/m^2^, mean (SD), range	30.7 (±5.4), 20.2–42.5
Blood pressure, mmHg	
Systolic, mean (SD), range	131.2 (±13.4), 98–157
Diastolic, mean (SD), range	73.2 (±9.0), 55–96
Pulse, BPM, mean (SD), range	68.4 (±10.7), 50–88
Smoking Status, *n* (%)	
Non-smoker	11 (26%)
Former Smoker	26 (62%)
Current Smoker	5 (12%)
Physical performance, mean (SD), range (Short Physical Performance Battery)	10.1 (±2.0), 4–12 (max score 12)
Cognitive performance, mean (SD), range (VA-SLUMS)	26.5 (±2.3), 22–30 (max score 30)
Quality of Life, mean (SD), range (Q-LES-Q-SF)	79.2% (±12.5%), 47%–100%

### 3.2 Higher VO2max correlates with less frailty, disability, and better quality of life

**FIGURE 2:**
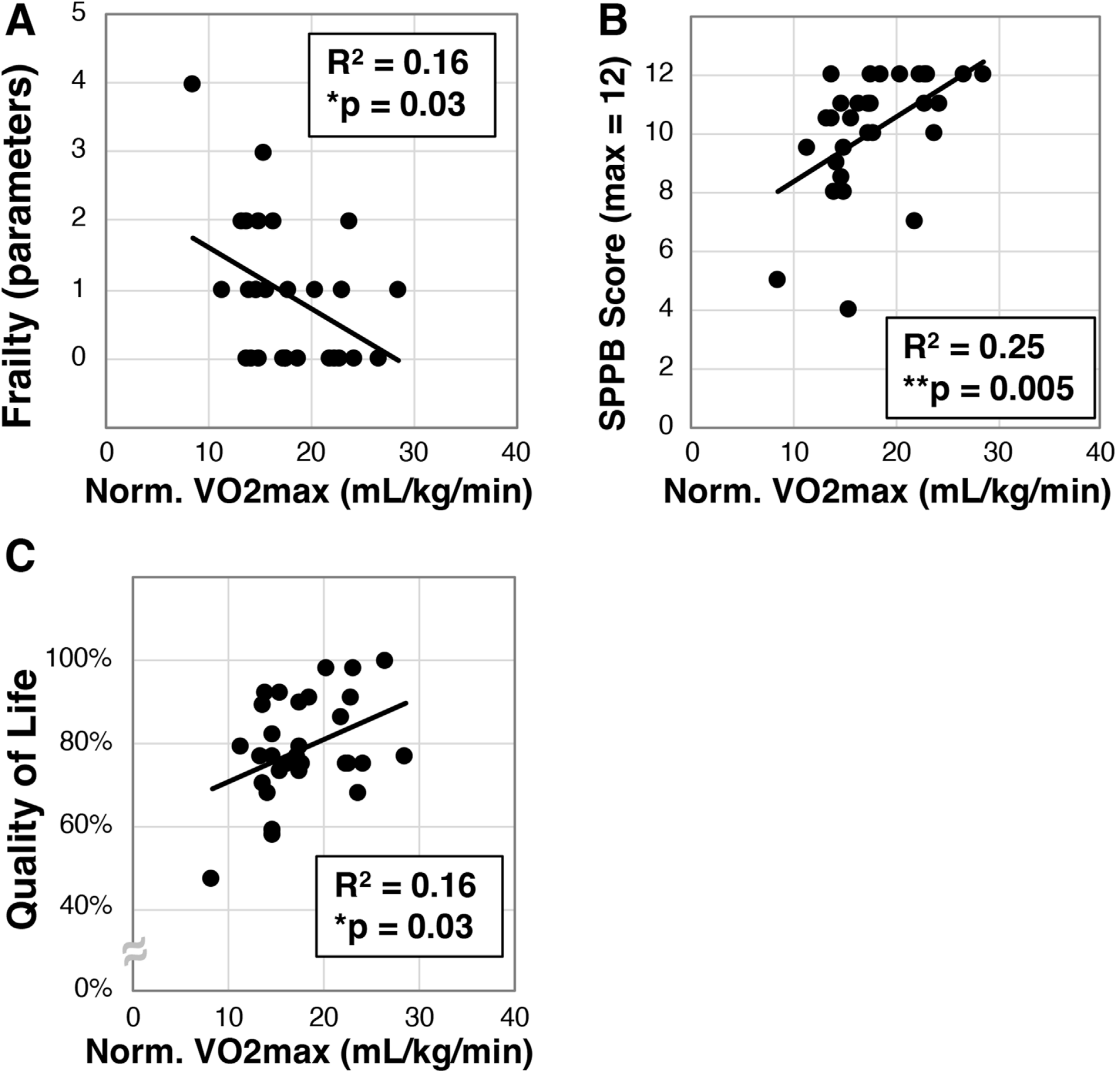
Analysis of maximal oxygen intake (VO2max) in older Veterans. VO2max was measured in a subset (N = 30) of the older Veterans in this study using an upright exercise bicycle. VO2max was then correlated to frailty **(A)**, disability as measured with the SPPB **(B)**, and quality of life measured with the Q-LES-Q-SF survey **(C)**. Statistical significance indicated by “*” indicates p < 0.05 and “**” indicates p < 0.01.

We next set out to determine if VO2max correlates with frailty in the older Veterans enrolled in our study. VO2max (mL/kg/min) was measured in a subset of participants (N = 30) on an upright cycle ergometer, and this cohort exhibited a mean VO2max of 17.9 ± 4.8 mL/kg/min. We next found a statistically significant correlation to frailty (*R* ^2^ = 0.16, *p = 0.03, Figure 2A). We also identified significant correlation of VO2max with the SPPB (R^2^ = 0.25, **p = 0.005, Figure 2B) and quality of life (R^2^ = 0.16, *p = 0.03, Figure 2C).

### 3.3 Greater 6-min walk correlates with VO2max, and quality of life

**FIGURE 3:**
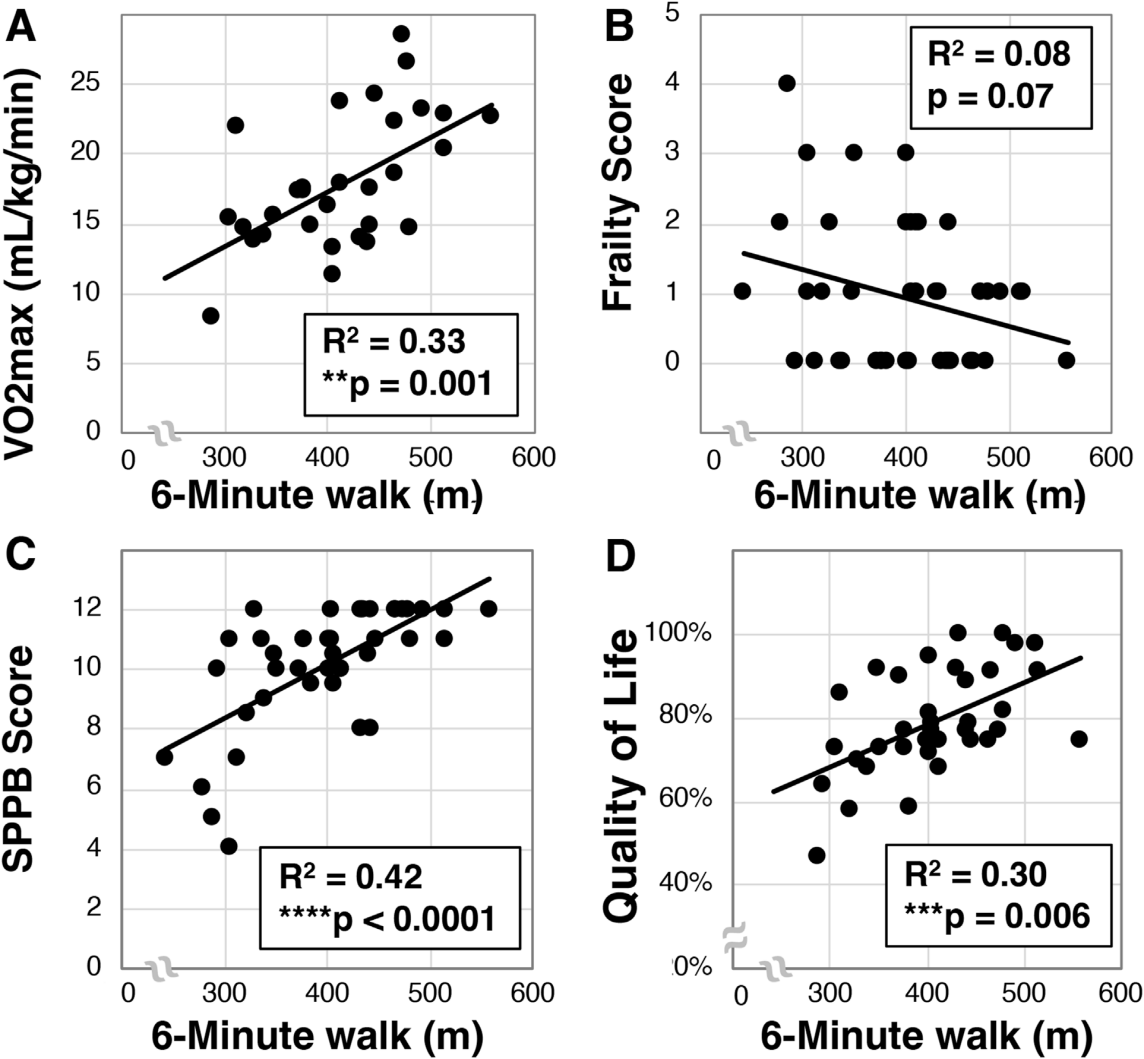
Analysis of 6-min walk in older Veterans. The 6-min walk was evaluated in older Veterans (N = 42) as the total distance covered over 6-min on a 15-m track. Total 6-min walk distance was then correlated to VO2max [**(A)**, N = 30], frailty **(B)**, SPPB **(C)**, and quality of life **(D)**. Statistical significance indicated by “**” as p < 0.01, “***” as p < 0.001, and “****” as p < 0.0001.

We then examined whether a relationship between 6-min walk and VO2max existed in our older Veteran cohort. We observed a mean 6-min walk distance of 397.8 ± 72.1 m (N = 42). Further, we found a statistically significant correlation between VO2max and 6-min walk (R^2^ = 0.33, **p = 0.001, N = 30, Figure 3A). Interestingly, despite correlation with VO2max, we only detected a trend towards correlation between the 6-min walk and frailty (R^2^ = 0.08, p = 0.07, N = 42, Figure 3B). We did observe a statistically significant relationship between 6-min walk and SPPB scores (R^2^ = 0.42, ****p < 0.0001, N = 42, Figure 3C) as well as quality of life (R^2^ = 0.30, ***p < 0.006, N = 42, Figure 3D).

### 3.4 Dominant arm strength correlates with frailty, but not disability or quality of life

**FIGURE 4:**
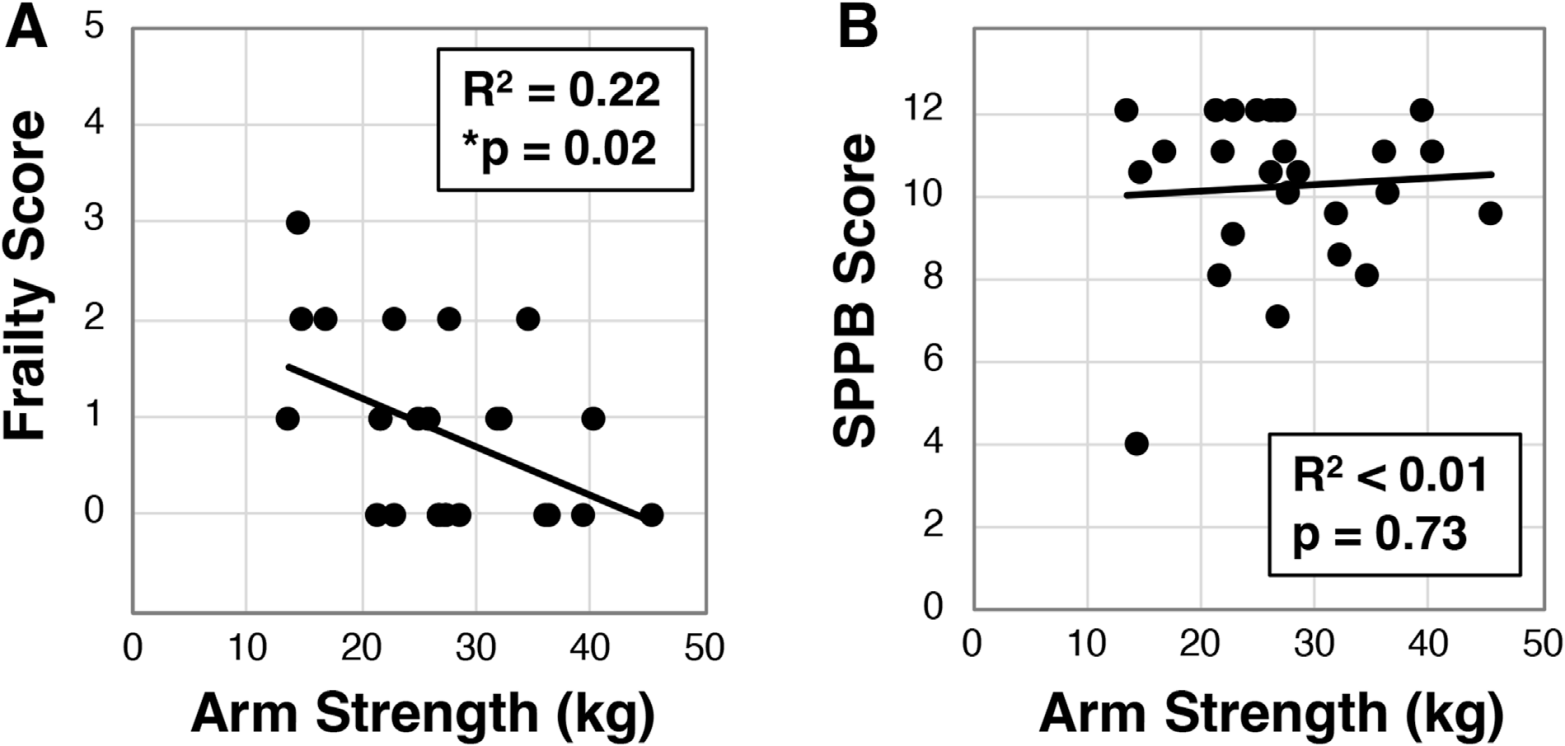
Analysis of dominant arm strength in older Veterans. Dominant arm strength was measured with participants (N = 26) attempting an arm curl while the arm was held down with a hand dynamometer. The best of three attempts was used to correlate to frailty status **(A)** or SPPB **(B)**. Statistical significance indicated by “*” as p < 0.05.

Using a handheld dynamometer, we next assessed arm and leg strength in a subset of participants (N = 27) and evaluated the relationship to other factors. Across the cohort we identified dominant arm strength was 28.8 ± 8.3 kg, while right leg strength was 29.8 ± 8.7 kg and left leg strength was 28.4 ± 8.4 kg. We further found that dominant arm strength inversely correlated to frailty (R^2^ = 0.22, *p = 0.02, Figure 4A), but surprisingly did not associate with SPPB (R^2^ < .01, p = 0.73, Figure 4B) or quality of life (Supplementary Figure S1). These findings were similar for left and right legs (Supplementary Figure S1). We did observe that dominant hand grip strength correlated with leg strength (p < 0.002 for both left and right, Supplementary Figure S1), but only trended for arm strength (p = 0.05, Supplementary Figure S1).

### 3.5 No difference in markers of serum inflammation in non-frail versus pre-frail/frail

**FIGURE 5:**
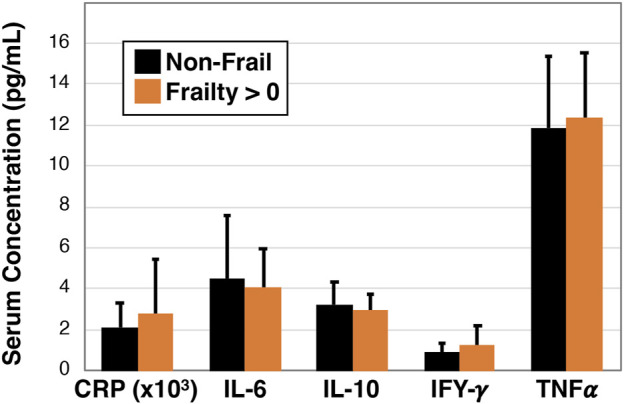
Inflammatory profile of non-frail and pre-frail/frail older Veterans. Serum was measured for specific cytokines using single and multiplex ELISA analysis and data were compared between non-frail (Frailty = 0 parameters, *n = 16*) and pre-frail or frail (Frailty > 0 parameters, *n* = 18) older Veterans. No statistically significant differences were observed.

To investigate whether blood biomarkers are associated with frailty we next examined C-reactive protein, interleukin-6, interleukin-10, interferon-gamma, and tumor necrosis factor alpha using serum based multiplex ELISA in non-frail participants (*n* = 16) and combined pre-frail and frail participants (*n* = 18, Figure 5). However, we did not detect a statistically significant association for any of the cytokines.

## 4 Discussion

Veterans are at greater risk of frailty and subsequent disability, morbidity, and mortality (Ganta et al., 2021; Orkaby et al., 2019). Thus, developing strategies for identifying frailty risk and pre-frailty may lead to early intervention and better health trajectories. Here we demonstrate that VO2max, 6-min walk, and muscle strength exhibit relationships with frailty status. Of particular interest, we found for the first time that dominant arm strength correlates with frailty (Figure 4A). Such a measure would be a useful surrogate for understanding frailty risk as the assessment can be performed in less than 2 min using a relatively inexpensive handheld device. This relationship might be explained by the close association between sarcopenia, the loss of muscle mass and function during aging, and frailty (Gielen et al., 2023), and the possibility that these measures of arm and leg strength might capture the sarcopenic state of the participants (Roberts et al., 2011; Wilkinson et al., 2018). Interestingly, although trending, arm strength did not correlate with grip strength. Grip strength alone is often looked at as an alternative to frailty assessment (Dudzinska-Griszek et al., 2017; Reeve et al., 2018; Spiegowski et al., 2022; Syddall et al., 2003), yet the predictive value of grip strength may be confounded by sex specific differences (Spiegowski et al., 2022). Future studies will be needed to understand if bicep strength may or may not be more informative than grip strength.

Additionally, we found that although arm strength correlated with frailty, it did not correlate with the short physical performance battery or quality life, nor other measures including VO2max and 6-min walk. Although these measures are all physically based, the lack of correlation among them – yet each having correlation with frailty – may be indicative of the multifactorial and multisystem nature of frailty. If this is the case, then perhaps these different measurements might provide different insights into an individual’s frailty. VO2max assessment is the gold standard for cardiovascular fitness, yet interestingly there are few reports that VO2max correlates with frailty (Fung et al., 2021; Olson et al., 2023). These reports used a deficit accumulation scale and the FRAIL scale for assessment, and our finding appears to be the first to directly find a correlation between VO2max and frailty in Veterans and as assessed by the Fried *et al.* based tool (Fried et al., 2001; Drey et al., 2011). Although VO2max assessment would be more difficult to implement than a typical frailty screen, these data add value in identifying potential biomarkers and underlying physiology contributing to frailty. Of further interest, Meijer *et al.*, have published a survey-based alternative to VO2max assessment that might be used in future research to evaluate capacity to predict frailty status (Meijer et al., 2022).

Alternatively, the 6 min walk can be easier to administer than a VO2max assessment, particularly for older adults – and its use to replace VO2max has been previously examined (Ross et al., 2010). Here we demonstrate in a Veteran cohort that the 6 min walk recapitulates many correlations found with VO2max, including the SPPB, quality of life, and a trend towards frailty. Thus, these findings support the 6 min walk as a tool that can inform across a wide range of health/functional parameters. However, there are some challenges that persist for a 6-min walk including the need for space and that the test may be strenuous for older adults and in the setting of co-morbidities. Some investigators are examining alternatives to the 6-min walk including a 3-minute stepper test (Balfour-Lynn et al., 1998; Bohannon et al., 2015) and 3-min walk test (Shigematsu et al., 2002). Future work should explore the relationship between these alternatives and frailty.

Unexpectedly, we did not find differences in serum inflammatory cytokines between non-frail and pre-frail/frail Veterans. The relationship between C-reactive protein and cytokines such as interleukin-6 with frailty has been nicely reviewed by Velissaris et al. (2017) *,* wherein they found a relationship in a majority of reviewed studies. That we did not identify a difference may be due to some study limitations. In particular, due to the COVID pandemic, we were only able to assess VO2max, muscle strength, and inflammatory cytokines on subsets of the participants. None of the measured cytokines exhibited significant differences between non-frail and pre/frail participants. We do not feel this was secondary to inadequate power, but possibly because we recruited participants interested in an exercise study and thus comprised a generally healthier cohort. As such, there may have been ceiling effects that masked the predictive potential of serum inflammatory biomarkers, particularly as we had only one participant with a frailty score of 4 or 5. An additional limitation of the study is that we were not able to recruit sufficient numbers of female Veterans that would have otherwise allowed deeper analysis of sex specific impacts that have been known to confound some measures [e.g., grip strength (Spiegowski et al., 2022)].

Overall, this study adds to our understanding of frailty in Veterans. We observed 47.6% of our cohort as pre-frail and 9.5% as frail, which is in line with a systematic review of over twenty-one community dwelling older adult studies that found 44.2% pre-frailty and 9.9% frailty overall (Collard et al., 2012). However, as noted above, our study may understate the prevalence of frailty in Veterans due to a potentially healthier cohort, despite only 26% of our participants had never smoked as opposed to 39% of Veterans and 58% of non-Veterans in general (Boersma et al., 2021). Interestingly, smoking status was not correlated with frailty in this study. Although lack of power may explain this outcome, this might also be due to the lack of specificity of our question that asked if the participant was either a non-smoker, former smoker, or current smoker. It would not, however, differentiate between an individual that was a former smoker of 1 year versus 30 years, for example,. Further study is needed to better understand the relationship between smoking status and frailty, particularly considering the higher rates of smoking in Veteran populations (Boersma et al., 2021; Prevention et al., 2018). Finally, this study also marks the first investigation of the relationship between VO2max, 6-min walk, and muscle strength assessments in Veterans. Knowledge of these relationships might be useful for understanding factors affecting frailty risk and the ability to recover from surgery or other interventions (Gillis et al., 2022; Gritsenko et al., 2020).

Limitations for this study to consider include a smaller sample size that ranged between 26 and 42 participants depending on the measure. The smaller sample size may have limited our ability to identify weak correlations. Ultimately, replication studies with larger sample sizes would help confirm the robustness of the findings from this study. Additionally, the use of Pearson’s correlations might bias findings by ignoring the possibility of other covariates that may confound statistical interactions. We therefore note the limitation that our smaller sample size does not permit the ability to investigate variables that may have influenced our findings. In particular, our cohort exhibited a mean BMI of 30.7, indicating a tendency towards obesity, which is also associated with low gait speed and frailty (Jayanama et al., 2022; Sagat, 2024), and therefore should this limitation should be considered when interpreting these results. Finally, we also note that the likelihood for correlation of different outcomes to frailty and to short physical performance battery may be biased by the sharing of the gait speed parameter. However, despite the use of a similar parameter (3 m walk for SPPB and 4 m walk for frailty), we identified differential correlation coefficients across our measures, suggesting significant contribution from the non-gait speed parameters of these indices.

## 5 Conclusion

This study investigated frailty in a cohort of older Veterans and the relationships to cardiorespiratory fitness and strength. Objectively measured cardiorespiratory fitness was associated with improved short physical performance battery scores, better quality of life, and lower frailty. The 6-min walk test, which is considered less burdensome than a VO2max assessment, was also found to recapitulate similar relationships as VO2max including positive correlation with SPPB and quality of life but did not correlate with frailty. Additionally, dominant arm strength and leg strength each negatively correlated with frailty, but the relationship to other indicators of physical performance was unclear. Data from this study suggest these measures of functional capacity have important relationships with frailty in Veterans and support the prospect that concerted interventions to promote safe and effective exercise training may maintain function and reduce frailty in older adults and Veterans.

## Data Availability

The original contributions presented in the study are included in the article/Supplementary Material, further inquiries can be directed to the corresponding authors.

## Supplementary material

SUPPLEMENTARY FIGURE S1 Correlation chart across measures. Correlative analysis was performed across demographic, functional, and cognitive assessments. For each comparison, correlation coefficient (r), p-value, and number compared are presented.
