# Supplementary figures and images for: VO2MAX, 6-minute walk, and muscle strength each correlate with frailty in US veterans
Source: Front Physiol. 2024 Sep 13;15:1393221. doi: 10.3389/fphys.2024.1393221 (PMC11427282; doi:10.3389/fphys.2024.1393221)

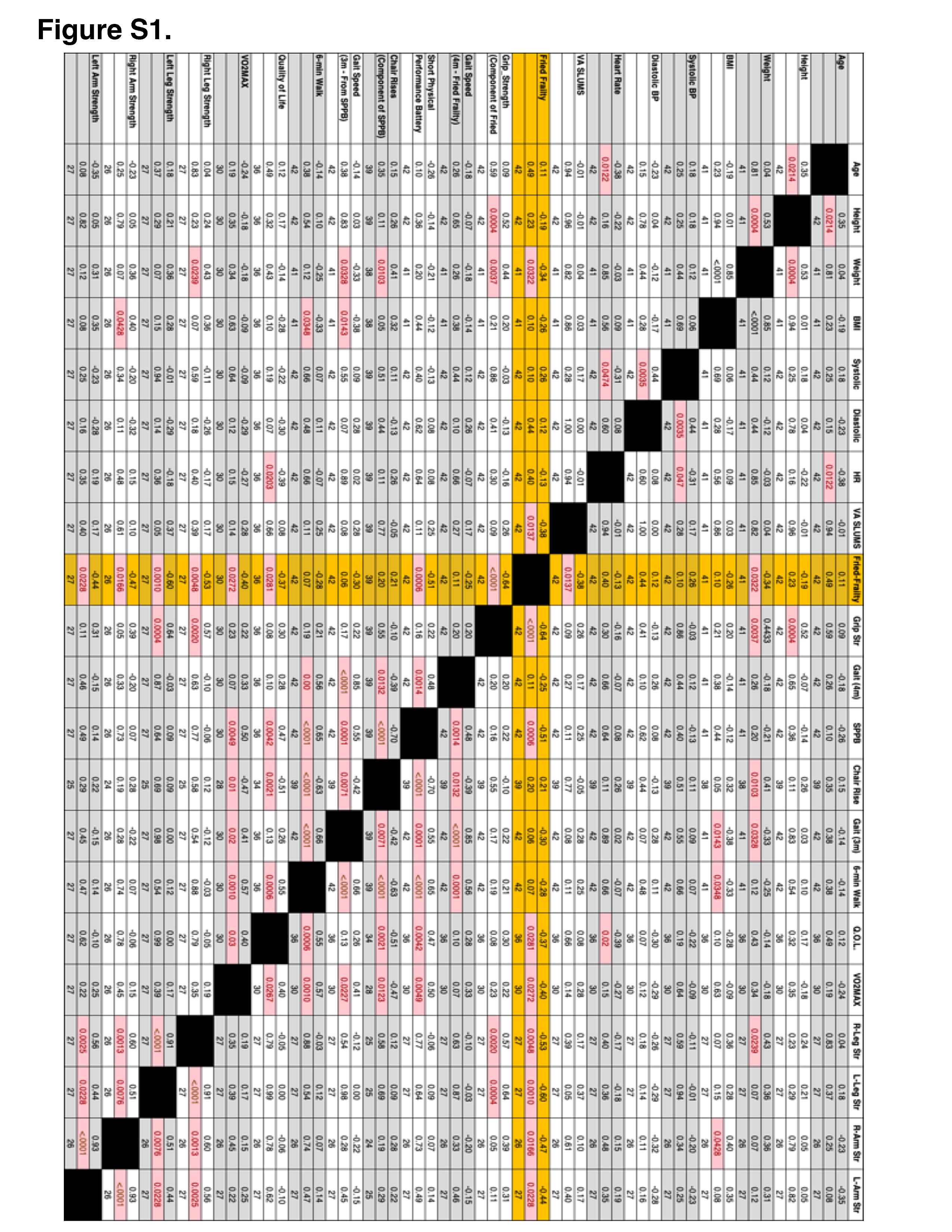

Supplement: Supplementary file 1 [file Image1.TIF]
